# The prevalence and factors associated with alcohol use disorder among people living with HIV/AIDS in Africa: a systematic review and meta-analysis

**DOI:** 10.1186/s13011-020-00301-6

**Published:** 2020-08-24

**Authors:** Mogesie Necho, Asmare Belete, Yibeltal Getachew

**Affiliations:** 1 Wollo University, College of Medicine and Health Sciences, Department of Psychiatry, Dessie, Ethiopia; 2 College of Medicine and Health Sciences, Department of Psychiatry, Diredawa University, Diredawa, Ethiopia

**Keywords:** Meta-analysis, Alcohol use disorder, AIDS, Africa

## Abstract

**Background:**

Alcohol use disorder (AUD) in HIV/AIDS patient’s decreases adherence and effectiveness of medications and help-seeking to HIV/AIDS care and treatment. This study, therefore, assessed the average 1 year prevalence and associated factors of alcohol use disorder in HIV/AIDS patients.

**Methods:**

We did an electronic data search on PubMed, Scopus, EMBASE, Psych-INFO libraries, African index Medicus and African Journals Online (AJOL). Google scholar was also investigated for non-published articles. The reference lists of published articles were also reviewed. The stata-11meta-prop package was employed. Subgroup and sensitivity analyses were done. Cochran’s Q-statistics and the Higgs I^2^ test were used to check heterogeneity. Publication bias was evaluated with Egger’s test and funnel plots.

**Results:**

Of 1362 articles identified using the search strategies; only 22 studies were included in the final analysis. The average 1 year prevalence of AUD was 22.03% (95% CI: 17.18, 28.67). The average prevalence of AUD in South Africa (28.77%) was higher than in Uganda (16.61%) and Nigeria (22.8%). The prevalence of AUD in studies published before 2011, 2011–2015, and after 2015 was found to be 13.47, 24.93, and 22.88% respectively. The average prevalence of AUD among studies with a sample size > 450 was 16.71% whereas it was 26.46% among studies with a sample size < 450. Furthermore, the average prevalence of hazardous, harmful, and dependent drinking was 10.87, 8.1, and 3.12% respectively. Our narrative analysis showed that male sex, cigarette smoking, family history of alcohol use, missing ART medication, mental distress, khat chewing, low CD4 count, and low income were among the associated factors for AUD in people with HIV AIDS. On quantitative meta-analysis for associated factors of AUD, the AOR of being male, Cigarette smoking and khat chewing were 5.5, 3.95, and 3.34 respectively.

**Conclusion:**

The average 1 year prevalence of AUD in HIV/AIDs patients was high and qualitatively factors such as being Male, cigarette smoking, and khat chewing were associated with it. Therefore, clinical services for people living with HIV/AIDS should integrate this public health problem. Policymakers should also develop guidelines and implementation strategies for addressing this problem.

## Background

Alcohol can be defined as a psychoactive drug capable of producing physiological as well as psychological dependence. Its Harmful use is associated with tremendous health, social and economic consequences [1–3]. AUDs contribute to 3.8% of the burden of disease globally [4] The global burden of deaths due to alcohol outweighs the synergized burden of deaths from acquired immunodeficiency syndrome (AIDS), human immunodeficiency virus (HIV), tuberculosis and violence [3]. AUDs can be of harmful use, hazardous or dependence use [5]. Harmful alcohol use is a pattern of use in the control of the will of individuals, whereas hazardous use is a pattern of use with the risk of harmful social, physical, and mental consequences. Alcohol dependence is the most severe end of the AUD spectrum [4, 5].

The prevalence of AUD in patients with retroviral infection has been evidenced higher than people without retroviral infection or the general public [6]. A systematic review and meta-analysis study by Duko et al. 2019 [7] showed that the average worldwide prevalence of AUD among patients with HIV/AIDS was found to be 29.8%. Other earlier studies in Africa reported that the prevalence of AUD ranges from 17 to 39.4% in Nigeria [8–10], 1.4 to 33% in Uganda [11–14], 6.6 to 48.5% in South Africa [15–18], 5.4 to 33% in Kenya [19–22], and 14.8% in Zambia [23]. There exists a scarcity of average data on alcohol use disorders in people with HIV in other African countries despite the high disease burden with people living with HIV (PLWHA) [24].

In Ethiopia studies conducted so far implied that AUD among HIV/AIDS patients was documented to be 32.6% in Jimma university specialized hospital [25], and 31.8% in Hawassa university comprehensive hospital [26], 14.2% in Bishoftu general hospital [27], 24.8% in Assela teaching hospital [28]. Moreover, a systematic review and meta-analysis study of alcohol consumption in Ethiopia by Ayano et al.2019 [29] reported that the prevalence of hazardous alcohol consumption was 8.96%.

Factors found to be associated with AUDs in HIV/AIDS patients include male gender, psychological morbidity, smoking cigarettes, the Christian religion, lower education, peer pressure, parental modeling, and social pressures [9, 10, 30–32]. A study from Jimma, Ethiopia also reported that being male, smoking cigarettes, and psychological distress as the associated factors for AUDs [25]. Similarly, another study from Hawassa, Ethiopia found factors such as being male, poor social support, medication non-adherence, khat chewing, and cigarette smoking as having a significant association with AUD [26].

The impacts of AUDs among PLHIV include decreased help-seeking to HIV/AIDS care and treatment facilities [33], adherence problem to antiretroviral treatment (ART) drugs [3, 34–37], a decline of CD4 cells, increased load of the virus [36, 38], rapid HIV/AIDS disease progression and rising of opportunistic diseases [38–42], development of drug-resistant HIV strains [3, 36, 38], and finally contributes to premature death [43, 44]. A study in the USA approved that if an individual with HIV consumes alcohol once/week his life span will be shortened by 2 years and by 6.5 years if alcohol is consumed daily [45]. Besides, patients with AUD might afraid of interaction between ART drugs and alcohol so that, skipping ART drugs during alcohol intake [9, 34]. Risky sexual and non-sexual behaviors [44, 46–48] are also common in people with AUD.

Even though multiple studies have been performed and showed that retrovirally infected patients have a higher prevalence of AUD than the general population in Africa [7–23, 27, 28, 49–52], to date no published study in Africa per investigators knowledge reported the average prevalence and associated factors of alcohol use disorders in human immune-deficiency virus-infected patients. Knowledge of the average prevalence of AUD and detecting its associated factors would assist policy-makers and program implementers in deciding evidence-driven prevention and promotion and treatment activities in this area.

So, this systematic review and meta-analysis study is warranted to [1] review the existing pieces of evidence on alcohol use disorder and its associated factors and [2] to determine the 1 year average prevalence of AUD and associated factors among PLWHA in Africa.

## Methods

This Preferred Reporting Items for Systematic Reviews and Meta-analysis Protocols (PRISMA-P) 2015 [53] had been used as an important guideline during the current study.

### Data sources and search strategies

We conducted our search in the following libraries; EMBASE, Psych-Info and Scopus, PubMed, African Index Medicus, and AJOL with a systematic search approach. Our search was not delimited to a specific year’s limitation of the studies. We conducted our search in PubMed with the following key terms and words: (Prevalence OR epidemiology OR magnitude OR incidence) AND (Alcohol use disorder OR alcohol abuse OR alcohol use) AND (HIV OR human immunodeficiency virus OR AIDS OR PLWHA OR ART) AND (factor OR risk OR risk factor OR determinant) AND (Southern Africa OR Central Africa OR East Africa OR North Africa OR Western Africa OR Sub-Saharan Africa). Google scholar was also searched for non-published articles. The reference lists of included studies were far investigated adequately to further include unaddressed eligible literature. For further clarification of unclear ideas, we contacted the authors of the included articles.

### Inclusion criteria

All cross-sectional, case-control, and cohort studies that had reported the prevalence of alcohol use disorders and/or it’s associated with human immune-deficiency virus-infected patients in Africa were included. A research article was eligible for inclusion to the current study if it was an observational study (cross-sectional, cohort and case-control study), the outcome studied should be AUD and the factors associated with it, and must be conducted in people living with HIV/AIDS who are on ART. In addition to this, the article had to be published in the English language and the area of study must be in Africa.

### Exclusion criteria

During the screening and analysis stage of the current study; we excluded letters, previously done systematic reviews and meta-analysis studies, interventional studies, commentaries, and editorials. Duplicated studies were also excluded to avoid being doubled in the analysis.

#### Selection of studies for inclusion in the review

Articles retrieved from the search databases were stored, managed, and used in an EndNote reference manager. Two review authors (MN, AB) reviewed each study’s title and abstracts stored in an EndNote reference manager independently and any disagreements between them whether to include or exclude the study for analysis was solved through discussion with the third author (YG).

#### Data extraction and management techniques

Using a standardized data extraction form, three authors (MN, AB, and YG) extracted data from included studies independently. The following elements were components of the data extraction form. First author last name, year of publication, study setting, sample size, number of events, data on the prevalence of alcohol use disorder, the tool used for assessment, associated factors, and odds ratio (OR) with 95% confidence intervals (CI).

#### Quality assessment methods

Two review authors (MN and AB) assessed the quality of all included studies independently. Differences regarding the quality score of included studies between the above review authors were solved with evidence-based discussion and a third reviewer (MN). The modified version of the Newcastle-Ottawa scale (NOS) [54] was used as a guideline for quality appraisal of the included studies which are cohort in design [11, 17, 21, 22]. Representativeness and size of the sample, comparability between study subjects, ascertainment of alcohol use disorder symptoms, and statistical quality were the dimensions of NOS in assessing the quality of each study. The quality of cross-sectional studies included [8–10, 12–16, 18–20, 23, 27, 28, 49, 51, 52, 55] was also evaluated using the Johanna Briggs Institute (JBI) critical appraisal checklist for prevalence studies [56].

The dimensions of JBI checklist for quality appraisal of prevalence studies include; the appropriateness of sample frame, sampling procedure of participants, adequacy of the sample size, appropriateness in the description of study subjects and setting, appropriateness of data analysis, usage of valid methods used for the identification of the condition, reliability of measurement, appropriateness of statistical analysis, and adequacy of the response rate.

### Data synthesis and analysis

The qualitative data for the present study was done with a narrative description and systematic review. The average 1 year prevalence of AUD and the average odds ratios (OR) of associated factors were calculated through random-effects [57] and quality-effects models [58]. Heterogeneity between the included studies was investigated with both Cochrane’s Q- statistic and the Higgs I^2^ statistics. A Higgs-I^2^ value greater than 50% was considered as an implication of heterogeneity between studies [59].

Since the average prevalence of AUD was found to have significant heterogeneity in doing the analysis, a sub-group analysis was performed to explore the sources of heterogeneity. All statistical analyses had been performed using Meta-XL version 5.3 [60] and the STATA11 Meta-prop package [61]. Publication bias was assessed with funnel plot [62] and Eggers regression test.

## Results

### Identification of studies

**Fig. 1:**
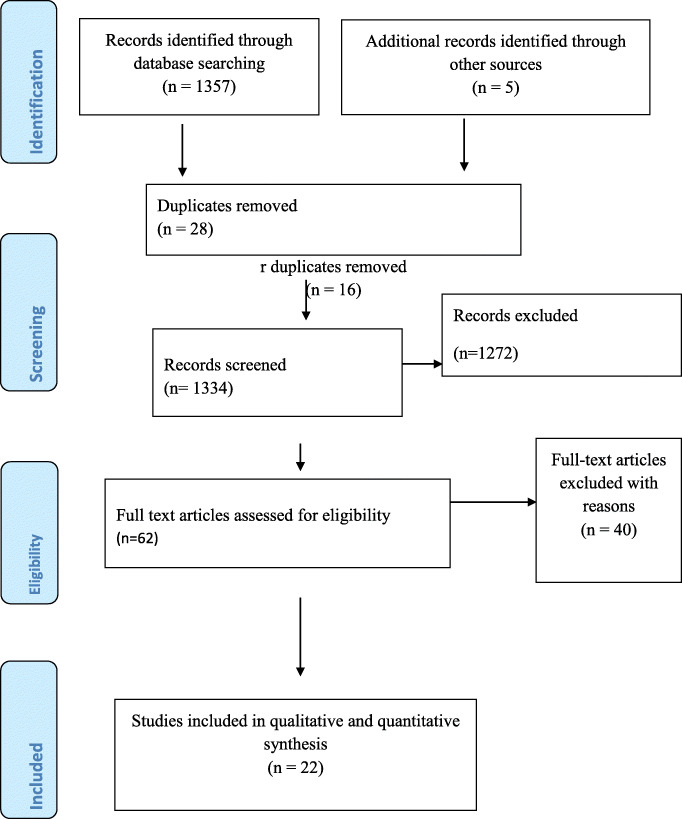
The PRISMA flow chart for the study

A total of 1357 articles were identified in our electronic search using the specified databases. A manual search for the reference lists other articles also resulted in 5 additional articles. This makes the total number of search results to be 1362 articles. Of these, 28 were duplicates and removed. A detailed screening of the remaining articles resulted in only 62 articles to be reviewed their full text for eligibility. Finally, only twenty-two articles were included in the analysis by fulfilling our pre-specified inclusion criteria (Fig. 1).

### Characteristics of included studies

A total of 22 studies in the Africa continent that investigated the 1 year prevalence of AUD in 16,774 patients on anti-retroviral therapy have been included in this systematic review and meta-analysis study [7–23, 27, 28, 49–52]. Based on the type of study design, 18 studies were cross-sectional type [8–10, 12–16, 18–20, 23, 27, 28, 49, 51, 52, 55] and the other four studies [11, 17, 21, 22] were cohort in design. Among the 22 studies in the meta-analysis [7–23, 27, 28, 49–52], 5 were from Ethiopia [7, 27, 28, 49, 51], 3 from Nigeria [8–10], 4 from Uganda [11–14], 4 form south Africa [15–18] and the remaining 6 studies were from Kenya, Namibia, and Zambia [19–23, 52].

Considering study publication, three were published before 2011 [9, 12, 15], eight were published b/n 2011–2015 [10, 13, 14, 19, 21–23, 51], and the remaining eleven were published after 2015 [7, 8, 11, 17, 18, 20, 27, 28, 49, 50, 52]. Ten of the included studies [8, 13–15, 18, 21–23, 27, 52] studied a sample of greater than 450 participants and the remaining 12 studies [7, 9–12, 16, 17, 19, 20, 28, 49, 51] studied a sample less than 450 participants (Table 1).

**Table 1:** Characteristics of studies on the 1 year prevalence alcohol use disorders among HIV/AIDS patients on ART which are incorporated in the narrative as well as meta-analysis according to author first name, year of publication, setting of study, design, sample size, assessment instrument, study population and magnitude of alcohol use disorder

Author, year	Study setting	Study design	Sample size	Assessment tool	Study population	AUD (%)	Number of cases with AUD (n)	Hazardous Drinker (%)	Harmful Drinker (%)	Dependent drinker (%)
Soboka et al. 2014 (1) [51]	Ethiopia	CS	401	AUDIT	HIV/AIDS patients	32.6	127	24.7(96)	2.8	5.1(20)
Egbe et al. 2017 (2) [8]	Nigeria	CS	1187	CIDI	HIV/AIDS patients	17	202	7.0	7.8	2.2
Goar et al. 2011 (3) [10]	Nigeria	CS	160	AUDIT	HIV/AIDS patients	39.4	63	10.6%	28.8	0
Farley et al. 2010 (4) [9]	Nigeria	CS	222	AUDIT	HIV/AIDS patients	12	47	NA	NA	NA
Bultum et al. 2018 (5) [27]	Ethiopia	CS	527	AUDIT	HIV/AIDS patients	14.2	75	10.8	2.5	0.8
Duko et al. 2019 (6) [55]	Ethiopia	CS	195	AUDIT	HIV/AIDS patients	31.8	62	NA	NA	NA
Segni et al. 2017 (7) [28]	Ethiopia	CS	418	AUDIT	HIV/AIDS patients	24.8	104	NA	NA	NA
Gebre. 2019 (8) [49]	Ethiopia	CS	332	AUDIT	HIV/AIDS patients	18.4	62	11.4	1.8	0.9
Hahn et al. 2018 (9) [11]	Uganda	Cohort	446	AUDIT	HIV/AIDS patients	30	133	NA	NA	NA
Wandera et al. 2015 (10) [13]	Uganda	CS	725	AUDIT	HIV/AIDS patients	33	293	NA	NA	NA
Martinez et al. 2008 (11) [12]	Uganda	CS	421	AUDIT	HIV/AIDS patients	1.4	6	NA	NA	NA
Chishinga et al. 2011 (12) [23]	Zambia	CS	649	AUDIT	HIV/AIDS patients	14.8	96	NA	NA	NA
Nakimuli et al. 2011 (13) [14]	Uganda	CS	500	AUDIT	HIV/AIDS patients	2	10	NA	NA	NA
Kibera et al. 2017 (14) [20]	kenya	CS	272	AUDIT	HIV/AIDS patients	14	38	NA	NA	NA
Kiunyu et al. (15)	Kenya	CS	164	AUDIT	HIV/AIDS patients	33	54	11	9.8	12.2
Cichowit et al.2017 (16) [17]	South Africa	Cohort	136	NA	HIV/AIDS patients	33	45	NA	NA	NA
Cerruti et al. 2016 (17)	South Africa	CS	1388	AUDIT	HIV/AIDS patients	6.6	93	NA	NA	0.3
Myer et al. 2008 (18) [15]	South Africa	CS	465	AUDIT	HIV/AIDS patients	27	126	NA	NA	NA
Morojele et al.2014 (19) [16]	South Africa	CS	303	AUDIT	HIV/AIDS patients	48.5	147	NA	NA	NA
Medley et al. 2014 (20) [21]	Namibia, Kenya, Tanzania	Cohort	3538	AUDIT	HIV/AIDS patients	15.7	184	NA	NA	NA
Tang et al.2019 (21) [52]	Namibia	CS	787	AUDIT	HIV/AIDS patients	30	237	NA	NA	NA
Seth et al. 2014 (22) [22]	Namibia, Kenya, Tanzania	cohort	3538	AUDIT	HIV/AIDS patients	5.4	184	0.2	3.2	2

Key: *CS* Cross-sectional, *AUDIT* Alcohol use disorder identification test, *ART* Anti-retroviral therapy, *CIDI* Composite International Diagnostic Interview

### Quality of included studies

In general, the summary quality assessment result of cohort studies included in the current meta-analysis ranges from 7 to 10 according to the scoring system of Newcastle Ottawa scale; one with moderate and three with good quality. The quality assessment for the remaining 18 cross-sectional studies based on the JBI checklist for prevalence studies ranges from 6 to 10; implying an appropriate methodological quality of the included studies.

### The average 1 year prevalence of alcohol use disorder among HIV/AIDS patients who were on antiretroviral therapy in Africa

**Fig. 2:**
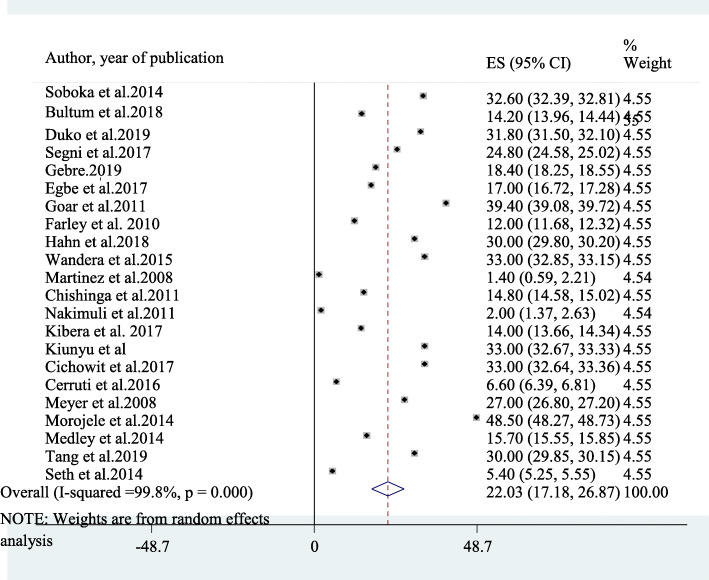
A forest plot for the prevalence of AUD in this study

Twenty-two studies had been included in the final meta-analysis to determine the average prevalence of AUD among patients on antiretroviral therapy in Africa [7–23, 27, 28, 49–52]. The reported prevalence of AUD among included studies in this review and meta-analysis study ranges from as low as 1.4% in Uganda [12] to as high as 48.5% in South Africa [16]. The average prevalence of AUD among patients on antiretroviral therapy in Africa using the random effect model was 22.03% (95% CI: 17.18, 28.67). This average prevalence has been influenced by a significant heterogeneity (I^2^ = 99.8%, *p*-value < 0.001) from the difference between the incorporated studies (Fig. 2).

### The 1 year prevalence of alcohol use disorders among HIV/AIDS patients based on country of origin and year of the study

Since the average prevalence of AUD was influenced by a significant heterogeneity during the analysis, a subgroup analysis has been implemented based on the country where the study was conducted, year of publication of the study, and sample size used in the study. Based on this among the 22 studies integrated with the meta-analysis [7–23, 27, 28, 49–52], 5 were from Ethiopia [7, 27, 28, 49, 51], 3 were from Nigeria [8–10], 4 were from Uganda [11–14], another 4 were from South Africa [15–18] and the remaining 6 studies were from Kenya, Namibia, and Zambia [19–23, 52].

The average prevalence of AUD among patients on ART in Ethiopia was 23.36% (95% CI: 17.53, 31.19) with (I^2=^ 98.6%, *p*-value < 0.001). The average prevalence of AUD in South Africa was also found to be 28.77% (95% CI: 10.39, 47.16) with (I^2^ = 99.2%, *p* < 0.001). On the other hand, the average 1 year prevalence of AUD in Uganda and Nigeria were 16.61% (95% CI: 6.86, 26.36) (I^2^ = 99.8%, *p* < 0.001) and 22.8% (95%CI: 6.83, 38.77) (I^2^ = 99.5%, *p* < 0.001) respectively.

**Fig. 3:**
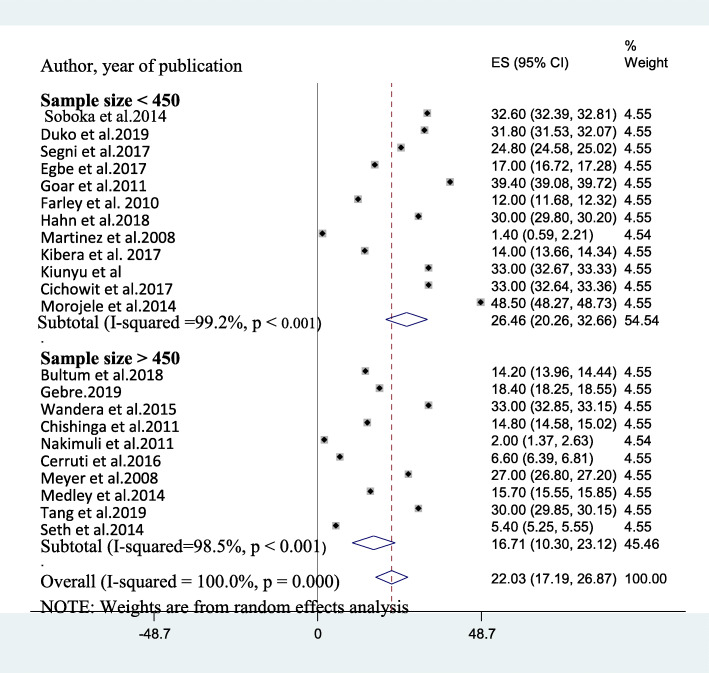
A sub-group analysis of AUD based on sample size

Considering year of publication, the average prevalence of AUD in studies published before 2011 [9, 12, 15], 2011–2015 [10, 13, 14, 19, 21–23, 51], and after 2015 [7, 8, 11, 17, 18, 20, 27, 28, 49, 50, 52] was found to be 13.47% (95%CI: 0.20, 26.75), 24.93% (95% CI: 15.10, 34.77) and 22.88% (95% CI: 17.71, 28.25) respectively. The average prevalence of AUD among studies which utilized a sample size > 450 [8, 13–15, 18, 21–23, 27, 52] was also obtained to be 16.71% (95% CI: 10.30, 23.12) (I^2^ = 98.5%, *p*-value < 0.001) whereas it was 26.46% (95% CI: 20.21, 32.72) (I^2^ = 99.20%, p-value< 0.001) among studies that utilized sample size < 450 [7, 9–12, 16, 17, 19, 20, 28, 49, 51] **(**Fig. 3**)** and (Table 2).

**Table 2:** A subgroup analysis of the 1 year prevalence of alcohol use disorder among HIV AIDS patients on ART in Africa with its 95% confidence interval

Subgroup		Number of studies	Estimates		Heterogeneity		
			Prevalence (%)	95% CI	I^2^	Q (DF)	*P*-value
Country	Ethiopia	5	23.36	17.53, 31.19	98.6%	195.17(4)	*P* < 0.001
	Nigeria	3	22.8	6.83, 38.77	99.5%	2037.2(2)	*P* < 0.001
	Uganda	4	16.61	6.86, 26.36	99.8%	2120 (3)	*P* < 0.001
	South Africa	4	28.77	10.39, 47.16	99. 2%	1246.23(3)	P < 0.001
	Kenya, Namibia & Zambia	6	18.82	10.09, 27.54	97.8%	123.2(5)	*P* < 0.001
Study design used	Cross-sectional	18	22.25	17.13, 27.37	98.8%	921.57(17)	*P* < 0.001
	Cohort	4	21.02	9.26, 32.79	99.6%	742.86 (3)	*P* < 0.001
Sample size studied	< 450	12	26.46		99.2%	1242.12 (11)	*P* < 0.001
	> 450	10	16.71	10.30, 23.12	98.5%	735.25(9)	*P* < 0.001
Year of publication	Before 2011	3	13.47	0.20, 26.75	96.5%	108.32(2)	*P* < 0.001
	2011–2015	8	24.93	15.10, 34.77	97.9%	135.8(7)	*P* < 0.001
	After 2015	11	22.88	17.71, 28.25	99.8%	2120 (10)	*P* < 0.001

Key; *DF* Degree of Freedom, *CI* Confidence Interval

### The average 1 year prevalence of hazardous alcohol use among HIV/AIDS patients who are on antiretroviral therapy in Africa

**Fig. 4:**
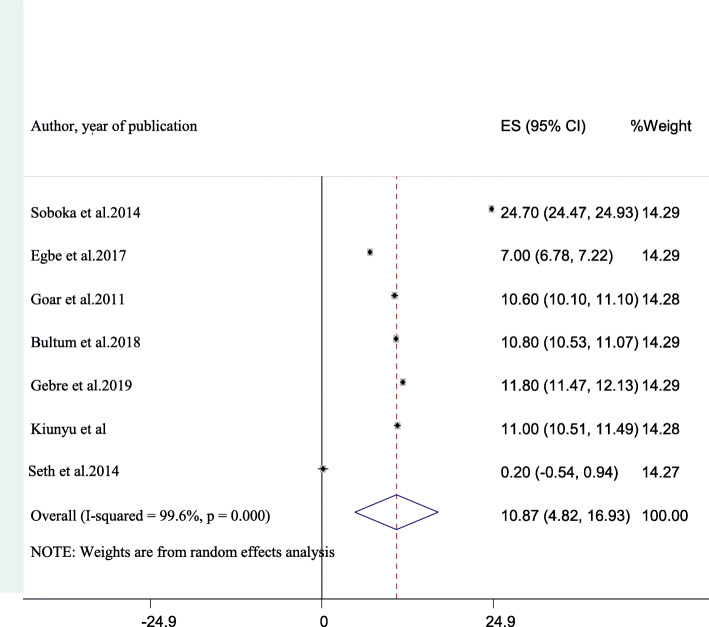
A forest plot for hazardous alcohol use in this study

Among the 22 studies included in the final analysis, data regarding hazardous drinking was described in seven studies [8, 10, 19, 22, 27, 49, 51]. The aggregate prevalence of hazardous drinking in these studies was 10.87% (95% CI: 4.82, 16.93). This average result was with considerable heterogeneity (I^2^ = 99.6%, *p*-value < 0.001) (Fig. 4).

### The average 1 year prevalence of harmful alcohol use among HIV/AIDS patients on antiretroviral therapy in Africa

**Fig. 5:**
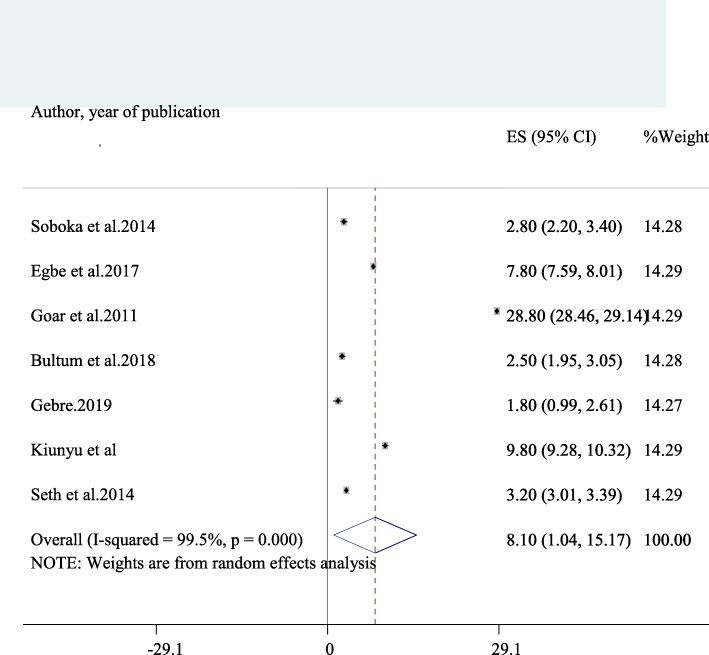
A forest plot for harmful alcohol use in this study

Seven studies reported data on the prevalence of harmful drinking in HIV/AIDS patients [8, 10, 19, 22, 27, 49, 51]. The average prevalence of harmful drinking among these studies was obtained to be 8.1% (95% CI: 1.04, 15.17) and was having a significant heterogeneity (I^2^ = 99.5%, *p*-value < 0.001) (Fig. 5). Consequently, we performed a subgroup of harmful drinking based on the sample size used. The Prevalence of harmful drinking among studies that used relatively larger sample (> 400) [8, 22, 27, 51] was found to be 4.08 (95% CI: 1.14, 7.02) whereas it was 13.47% (− 2.97, 29.91) in studies which used sample size < 400 [10, 19, 49].

### The average 1 year prevalence of dependent drinking among HIV/AIDS patients who are on antiretroviral therapy in Africa

**Fig. 6:**
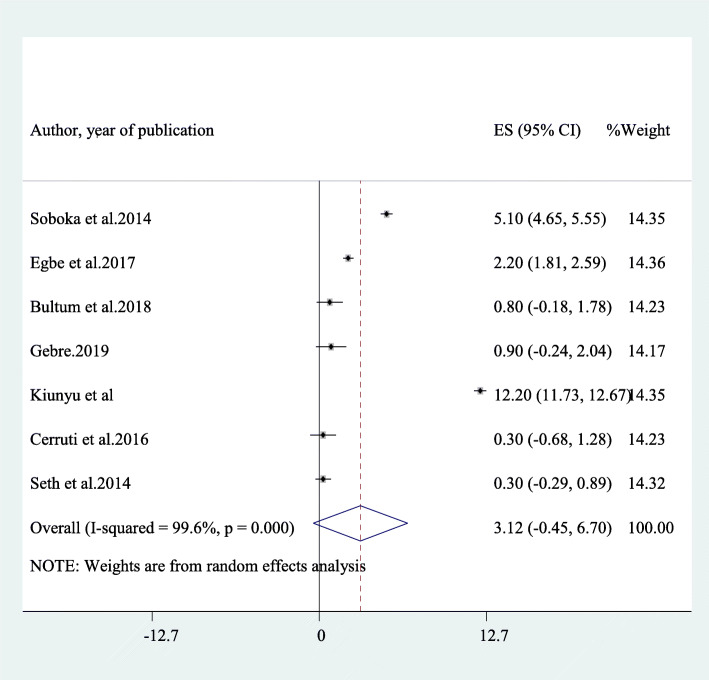
A forest plot for dependent alcohol use in this study

Seven other studies [8, 10, 19, 22, 27, 49, 51] also reported dependent drinking in HIV/AIDS patients on antiretroviral therapy. The average prevalence of dependent drinking in these studies was 3.12% (95% CI: 1.45, 6.70) and an obvious heterogeneity has also been detected in the result (I^2^ = 99.6%, *p*-value < 0.001) (Fig. 6). The average prevalence of dependent drinking among studies that utilized a sample of more than 400 [8, 18, 22, 27, 51] was 1.76% (1.16, 3.68) whereas it was 6.56% (95% CI: 2.51,17.64) among smaller sample studies [19, 49].

### Sensitivity analysis

To detect further the source of heterogeneity that influences the average 1 year prevalence of AUD, we also did one study leave out at a time sensitivity analysis. The result from the sensitivity analysis revealed that the average estimated prevalence of AUD obtained when each study was left out from analysis was within the 95% confidence interval of the average prevalence of AUD when all studies were run together. Therefore, the result of the average prevalence of AUD in HIV patients was not influenced by a single particular study. Moreover, the sensitivity analysis result revealed that the average AUD prevalence ranges between 20.77 (95% CI: 16.33, 25.31) and 22.98% (95% CI: 18.05, 27.91) when each study was excluded (Table 3).

**Table 3:** a sensitivity analysis of the 1 year prevalence of alcohol use disorder among HIV AIDS patients on ART in Africa when each indicated studies are removed at a time with its 95% confidence interval

No	Study excluded	Prevalence of Alcohol use disorder	95% Confidence interval
1	Soboka et al. 2014 [51]	21.53%	16.53, 26.52
2	Egbe et al. 2017 [8]	22.27%	17.25, 27.29
3	Bultum et al.	22.4%	17.39, 27.41
4	Duko et al. 2019 [55]	21.56%	16.58, 26.55
5	Segni et al. 2017 [28]	21.9%	16.82, 26.97
6	Gebrie, 2019 [49]	22.20%	17.02, 27.38
7	Goar et al. 2011 [10]	21.20%	16.31, 26.10
8	Farley et al. 2010 [9]	22.51%	17.54, 27.48
9	Hahn et al. 2018 [11]	21.65%	16.60, 26.69
10	Wandera et al. 2015 [13]	21.51%	16.52, 26.49
11	Martinez et al. 2008 [12]	23.01%	18.07, 27.95
12	Chishinga et al. 2011 [23]	22.37%	17.34, 27.40
13	Nakimuli et al. 2011 [14]	22.98%	18.05, 27.91
14	Kibera et al. 2017 [20]	22.41%	17.43, 27.40
15	Kiyunyu et al.	21.51%	16.53, 26.48
16	Cichiwoti et al. 2017	21.51%	16.54, 26.48
17	Cerruti et al. 2016	22.76%	17.94, 27.59
18	Myer et al. 2008 [15]	21.79%	16.71, 26.88
19	Morojele et al. 2014 [16]	20.77%	16.33, 25.31
20	Medley et al. 2014 [21]	22.33%	17.21, 27.45
21	Tang et al. 2019 [52]	21.65%	16.55, 26.75
22	Seth et al. 2014 [22]	22.82%	18.30, 27.34

### Publication bias

**Fig. 7:**
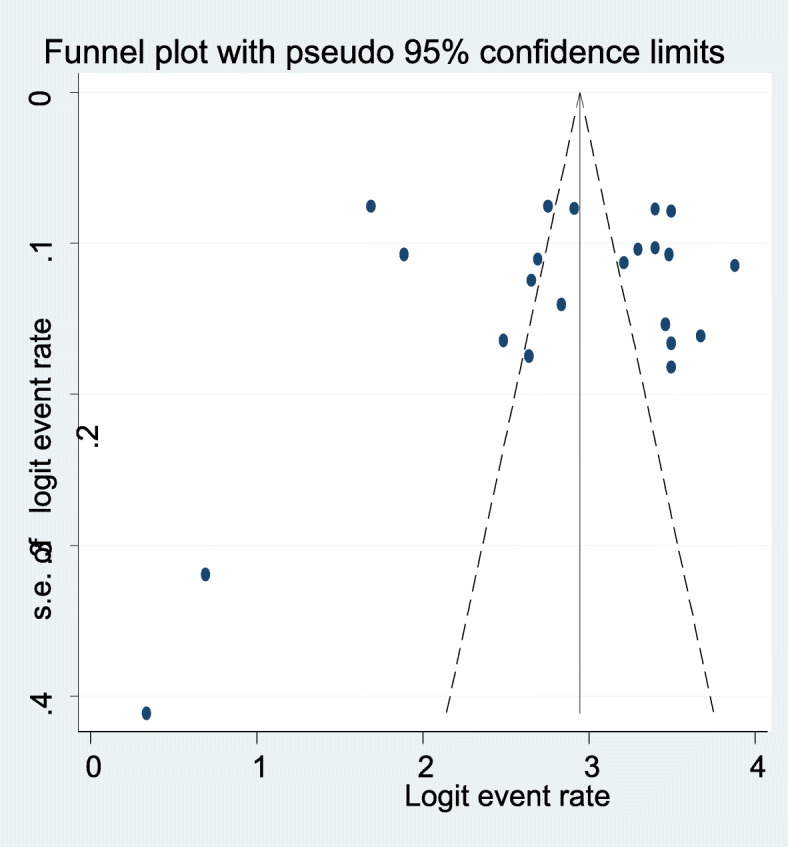
A funnel plot of publication bias for alcohol use disorder in this study

We carried out an Egger’s publication bias plot to detect the presence of publication bias but it is near the origin and the result of Eggers publication bias plot had insignificant *p*-value (*P* = 0.22), on condition that no substantial publication bias for the prevalence AUD in Africa. Moreover, a visual inspection from a funnel plot for a Logit event rate of prevalence of AUD in HIV AIDS patients against its standard error suggests additional evidence for the absence of a small study effect (Fig. 7**)**.

### Narrative description of the associated factors for 1 year prevalence of alcohol use disorders

Of 22 included studies, 12 studies that reported associated factors for AUD among HIV AIDS patients were included in our narrative analysis [8–10, 16, 17, 20, 21, 27, 28, 49, 51, 55] (Table 4). Seven of the included studies [7, 9, 10, 16, 28, 49, 51] reported an association between being male and AUD. Cigarette smoking was also reported as a related factor for AUD in four [7, 27, 49, 51] studies. Family history of alcohol use [27, 28], missing ART medication [21, 27], mental distress [51], khat chewing [7, 27, 49], educational status [10, 27], low CD4 count [49], low income [10], orthodox religion [51], protestant religion [51] had also a strong and significant association with AUD in people with HIV AIDS in Africa.

**Table 4:** Characteristics of associated factors for the 1 year prevalence alcohol use disorder among HIV AIDS patients in Africa by their Odds ratio, Confidence interval, association strength, author and year of publication

Associated factors	Odds ratio (AOR)	95% CI	Strength of association	Author, year of publication	Remark
Lower ducational status	8.5	1.70,42.99	Strong and positive	Bultum et al. 2018 [27]	
Cigarette smoking	3.49	1.01,12.13	Strong and positive	Bultum et al. 2018 [27]	
Chat chewing	5.11	1.60,16.33	Strong, positive	Bultum et al. 2018 [27]	
Family history of alcohol use	3.58	15.2,8.47	Strong and positive	Bultum et al. 2018 [27]	
Missing ART drugs	3.05	1.30,7.12	Strong and positive	Bultum et al. 2018 [27]	
Being Male	3.48	1.27, 9.59	Strong, positive	Gebrie, 2019 [49]	
Cigarette smoking	5.12	4.02, 8.61	Strong and positive	Gebrie, 2019 [49]	
Chat chewing	3.23	2.06, 6.89	Strong and positive	Gebrie, 2019 [49]	
CD4 count of 0–200	19.49	1.74, 218.4	Strong and positive	Gebrie, 2019 [49]	
Being male	14.1	5.84, 33.87	Strong and positive	Segni et al. 2017 [28]	
Family history of substance use	2.66	1.15, 6.13	Strong and positive	Segni et al. 2017 [28]	
Missing a dose of HIV medications	2.04	1.67, 2.49	Strong and positive	Medley et al. 2014 [21]	
Inconsistent condom use	1.49	1.23, 1.79	Moderate and positive	Medley et al. 2014 [21]	
Commercial sex	1.57	1.06, 2.32	Moderate and positive	Medley et al. 2014 [21]	
STI	1.40	1.10, 1.77	Moderate and positive	Medley et al. 2014 [21]	
Male sex	2.43	1.76, 5.76	Strong and positive	Duko et a; 2019 [55]	
Poor social support	1.34	1.12, 6.73	weak and positive	Duko et a; 2019 [55]	
Being medication non-adherent	1.78	1.33, 6.79	Strong and positive	Duko et a; 2019 [55]	
Chat chewing	1.67	1.16, 5.45	Strong and positive	Duko et a; 2019 [55]	
Cigarette smoking	3.76	2.16, 7.54	Strong and positive	Duko et a; 2019 [55]	
Male sex	*X*^2^ = 17.999	*P* = 0.000	Strong and positive	Goar et al. 2011 [10]	
Lower education	*X*^2^ = 9.86	*P* = 0.000	Strong and positive	Goar et al. 2011 [10]	
low income	*X*^2^ = 13.68	*P* = 0.002	Strong and positive	Goar et al. 2011 [10]	
Male sex	5.2	2.48, 11.22	Strong and positive	Farley et al. 2010 [9]	
Orthodox religion	2.3	1.22,4.31	Strong and positive	Soboka et al. 2016 [51]	
Protestant religion	2.3	1.23,4.34	Strong and positive	Soboka et al. 2016 [51]	
Male sex	2.23	1.30, 3.83	Strong and positive	Soboka et al. 2016 [51]	
Cigarette smoking	3.4	1.38–8.40	Strong and positive	Soboka et al. 2016 [51]	
Mental distress	2.24	1.40, 3.64	Strong and positive	Soboka et al. 2016 [51]	
Being female	0.1	0.05,0.19	Strong and negative	Egbe et al. 2017 [8]	Harmful alcohol use
Being Christian	3.44	1.43,8.27	Strong and positive	Egbe et al. 2017 [8]	Harmful alcohol use
Being female	0.32	0.13, 0.76	Strong and negative	Egbe et al. 2017 [8]	Dependent drinking

### The association between male sex and alcohol use disorder in HIV/AIDS patients

The association of being a male and higher risk of AUD in HIV/AIDS patients was reported in seven of the included studies [7, 9, 10, 16, 28, 49, 51]. The average adjusted odds ratio of the increased risk of having AUD was 5.5 (95% CI: 1.10, 9.98) (I^2^ = 90%, *P* < 0.01). This implied that male HIV/AIDS patients who were on ART were 5.5 times at higher risk of having alcohol use disorder as compared to female patients who were on ART therapy.

### The association between cigarette smoking and chat chewing with an alcohol use disorder

Among the 22 studies incorporated in the current meta-analysis [7–23, 27, 28, 49–52], four [7, 27, 49, 51] had reported cigarette smoking as an independent factor for AUD in HIV patients. The average adjusted odds ratio of cigarette smoking in these studies was found to be 3.95% (95% CI: 3.00, 4.89) (I^2^ = 96.2%, *P* < 0.01). This result suggested that patient’s on ART who were smoking a cigarette were on average 4 times at increased risk of developing AUD than patients who were not smoking a cigarette. Similarly, three of the above-indicated studies [7, 27, 49] had also reported khat chewing as a risk factor for AUD. The average adjusted odds ratio of khat chewing among these studies was found to be 3.34% (95% CI: 1.71, 4.96) (I^2^ = 98.2%, *P* < 0.01). This implied that patients who were chewing khat were on average 3.3 times more likely to have AUD than patients who were not chewing khat.

## Discussion

In general, AUD in people living with HIV/AIDS is highly prevalent and linked with non-adherence to antiretroviral therapy, decreased help-seeking, and health care utilization as well as poor HIV treatment outcomes [63]. Despite this and the presence of multiple single setting studies in Africa, to date as per the knowledge of investigators, this review and meta-analysis on the prevalence of AUD and its associated factors in patients who are on antiretroviral therapy are the first of its kind in Africa. This study, therefore, aimed and assessed the average 1 year prevalence of AUD and its associated factors in the African population, and the data obtained from this meta-analysis study would be significant evidence to future researchers, clinical practitioners, public health experts, and policymakers.

Twenty-two studies that assessed AUD in African HIV/AIDS patients were included in the current meta-analysis. The average prevalence of AUD among included studies was found to be 22.03%. This average prevalence of AUD was higher than the average prevalence of alcohol use disorder in the general population [29]. This difference might be caused because people with a chronic medical illness like HIV/AIDS, might engage in alcohol use as a coping strategy for a way for psychological distress and anxiety associated with the perceived severity of the illness and medication side-effects [64].

The average prevalence of AUD among PLWHA in the current study was lower as compared to the result of a systematic review and meta-analysis study by Duko et al.2019 [7]; which reported the average worldwide prevalence of AUD among HIV/AIDS patients to be 29.8%. The differences in economic, social, and cultural factors in which alcohol use behaviors are favorably higher in developed countries than African countries might cause variance. The average prevalence of AUD in the present study was comparatively lower than the prevalence of AUD in individuals with psychiatric disorders which ranges from 28 to 70% [65]. The poor judgment and insight in patients with psychiatric disorders could result in the elevated prevalence of AUD in this group of population.

This average prevalence of AUD in our study was considerably higher in South Africa (28.8%) than the average prevalence estimate in Uganda (16.6%). South Africa is a relatively economically advanced nation than Uganda so individuals would have the ability to afford alcohol.

Variation in culture, the ease of access and accessibility of alcoholic drinks, differences in the number of included studies in the analysis might also add to the difference in the prevalence of AUD among HIV/AIDS patients.

As expected, the pooled estimated prevalence of AUD in studies that studied a relatively higher sample (> 450) was significantly lower (16.7%) than the average estimated prevalence for studies which assessed smaller sample (< 450) which was 26.5%. This could be due to the decreases in the probability of a standard error when using a larger sample size and so providing a more precise and reliable result with strong power [66, 67].

Besides, the average estimated 1 year prevalence of AUD was higher among studies done in 2011 and after (22.9 to 24.9%) than the average estimate of AUD in studies that were done before 2011(13.5%). The increased availability of alcohol and alcohol advertising programs at current times could bring such variation.

The average estimated 1 year prevalence of hazardous alcohol use, harmful alcohol use, and dependent drinking in this study were found to be 10.87, 8.1, and 3.12% respectively. The prevalence of hazardous alcohol use is lower than alcohol use disorder in the present study. This could be due to the small number of studies included in the estimated prevalence of hazardous drinking that affected the precision of the estimate. The average estimated prevalence of AUD was 4% in studies that used a larger sample size (> 400) and is lower than the average estimated prevalence in studies that assessed a relatively smaller sample size (< 400) (13.5%). Furthermore, the estimated prevalence of dependent drinking was also higher in smaller sample studies (6.7%) than larger sample studies (1.8%).

Concerning the associated factors for AUD, being male [7, 9, 10, 16, 28, 49, 51], Cigarette smoking [7, 27, 49, 51], family history of alcohol use [27, 28], missing ART medication [21, 27], mental distress [51], khat chewing [7, 27, 49], educational status [10, 27], low CD4 count [49], low income [10], orthodox religion [51], protestant religion [51] had a strong and significant association with AUD in people with HIV AIDS in Africa.

Association between male sex and AUD was reported in this study. The average estimated odds ratio of being male as a risk factor for AUD was 5.5 in this study. This showed that male patients with HIV were 5, 5 times more vulnerable to develop AUD than female patients. Supportive evidence for this existed in a meta-analysis study [7]. Factors related to variation in neurochemistry could be responsible for this. This can be illustrated by a US study that revealed that a higher rate of dopamine release was observed in men than women despite the same level of alcohol intake [68] that can further reinforce the alcohol-seeking behavior and heighten the risk of AUD. Some cultures restrict alcohol consumption in women which could further reduce the risk of AUD in women and other factors in the environment may have an additive role for the difference [69].

The average estimated odds ratio of cigarette smoking and chat chewing in this study was 3.9 and 3.3. Patients who were smoking cigarettes and chewing chat were nearly 4 times and 3.3 times more likely to develop AUD than patients who were not smoking cigarettes and chewing khat. These substances have a similar mechanism of action with alcohol [70] and therefore one can potentiate the rewarding effect of the other [71].

### Difference between studies included in the current review and meta-analysis study

This meta-analysis study had a significant heterogeneity from the variance between the included studies. Therefore, we further perform a subgroup analysis to explore the source of heterogeneity. The subgroup analysis revealed that the country of origin for the study and the sample size used among included studies and study period was responsible for the variance in the prevalence of alcohol use disorder between included studies. On top of this, we conducted one study leave out sensitivity analysis but the result showed that the overall average estimated prevalence of AUD was not under the influence of a single particular study. This meta-analysis study on AUD has limitations to be considered in using its result. The primary limitation is that it was under the influence of significant heterogeneity. Furthermore, at some sub-groups, we integrate a few numbers of studies so that the precision of estimate might be affected.

### Implications of the findings to future research, clinical practice, and policy

This meta-analysis study has placed some important implications for future researchers, clinical practitioners, and policymakers. The high average prevalence of AUD among people living with HIV/AIDS in Africa obtained in the current study as compared to the average estimated 1 year prevalence of AUD in the general population urges future researchers to have an area of research for why and what determining factors were responsible for this. Secondly, the result of this study informed that clinical practitioners working in the health care facility especially those working on anti-retroviral treatment centers have to be aware of the problem and intervening regularly while managing such patients. Finally, policymakers and program planners need to give attention to this public health problem so that better ways of treatment and prevention approaches for the holistic implementation of alcohol use service of people living with HIV/AIDS with the existing health care services.

## Conclusion

This review and meta-analysis study found a high pooled magnitude of AUD in the African population living with HIV; nearly one in five (22%). This average estimated prevalence of AUD was subjected to substantial heterogeneity. Therefore, we did a sub-group analysis and it was found that country of origin for the study; sample size and year of the study were among the responsible factors for between studies variance. The average prevalence of AUD was considerably higher in South Africa (28.8%) than in Uganda (16.6%). The average estimated 1 year prevalence of AUD in studies with a higher sample (> 450) was lower (16.7%) than studies with a smaller sample (< 450) (26.5%). The average 1 year prevalence of AUD was also 13.47, 24.93, and 22.88% in studies published before 2011, 2011–2015, and after 2015 respectively.

Our narrative synthesis revealed that actors such as being male, Cigarette smoking, Khat chewing, family history of alcohol use, missing ART medication, mental distress, low CD4 count, and low income were some of the associated factors with AUD in people with HIV AIDS in Africa. Our quantitative meta-analysis for the associated factors of AUD in HIV/AIDS patients also showed that the AOR for the male sex, cigarette smoking, and khat chewing were 5.5, 3.95, and 3.34 respectively. Therefore, clinical service delivery to HIV/AIDS patients should primarily focus on early detection and integrated management of AUD and the aforementioned factors. Policymakers should also use the result of the current study as a baseline to develop appropriate policies and strategies which aids in the implementation procedures during the integration of management of AUD into the clinical service of people living with HIV/AIDS.

## Data Availability

All data regarding this research work is incorporated in the paper.

## Abbreviations

AIDS: Acquired Immune Deficiency Syndrome
AUD: Alcohol Use Disorder
ART: Anti-Retro viral Therapy
CI: Confidence Interval
HIV: Human Immune-Deficiency Virus
OR: Odds Ratio
PLWHIV: People Living with Human Immune-Deficiency Virus
PRISMA-P: Preferred Reporting Items for Systematic Reviews and Meta-analysis
USA: United States of America
WHO: World Health Organization
